# High-quality draft genome sequence of *Flavobacterium suncheonense* GH29-5^T^ (DSM 17707^T^) isolated from greenhouse soil in South Korea, and emended description of *Flavobacterium suncheonense* GH29-5^T^

**DOI:** 10.1186/s40793-016-0159-5

**Published:** 2016-06-16

**Authors:** Nisreen Tashkandy, Sari Sabban, Mohammad Fakieh, Jan P. Meier-Kolthoff, Sixing Huang, Brian J. Tindall, Manfred Rohde, Mohammed N. Baeshen, Nabih A. Baeshen, Alla Lapidus, Alex Copeland, Manoj Pillay, T. B. K. Reddy, Marcel Huntemann, Amrita Pati, Natalia Ivanova, Victor Markowitz, Tanja Woyke, Markus Göker, Hans-Peter Klenk, Nikos C. Kyrpides, Richard L. Hahnke

**Affiliations:** Department of Biological Sciences, Faculty of Science, King Abdulaziz University, Jeddah, Saudi Arabia; Leibniz Institute DSMZ – German Collection of Microorganisms and Cell Cultures, Braunschweig, Germany; HZI – Helmholtz Centre for Infection Research, Braunschweig, Germany; Center of Nanotechnology, King Abdulaziz University, Jeddah, Saudi Arabia; Centre for Algorithmic Biotechnology, St. Petersburg State University, St. Petersburg, Russia; Department of Energy Joint Genome Institute, Genome Biology Program, Walnut Creek, CA USA; Biological Data Management and Technology Center, Lawrence Berkeley National Laboratory, Berkeley, CA USA; School of Biology, Newcastle University, Newcastle upon Tyne, UK

**Keywords:** Aerobic, Gliding motility, Greenhouse soil, *Flavobacteriaceae*, *Bacteroidetes*, GEBA, KMG-1, Tree of Life, GGDC, Carbohydrate active enzyme, Polysaccharide utilization loci

## Abstract

*Flavobacterium suncheonense* is a member of the family *Flavobacteriaceae* in the phylum *Bacteroidetes*. Strain GH29-5^T^ (DSM 17707^T^) was isolated from greenhouse soil in Suncheon, South Korea. *F. suncheonense* GH29-5^T^ is part of the ***G****enomic****E****ncyclopedia of****B****acteria and****A****rchaea* project. The 2,880,663 bp long draft genome consists of 54 scaffolds with 2739 protein-coding genes and 82 RNA genes. The genome of strain GH29-5^T^ has 117 genes encoding peptidases but a small number of genes encoding carbohydrate active enzymes (51 CAZymes). Metallo and serine peptidases were found most frequently. Among CAZymes, eight glycoside hydrolase families, nine glycosyl transferase families, two carbohydrate binding module families and four carbohydrate esterase families were identified. Suprisingly, polysaccharides utilization loci (PULs) were not found in strain GH29-5^T^. Based on the coherent physiological and genomic characteristics we suggest that *F. suncheonense* GH29-5^T^ feeds rather on proteins than saccharides and lipids.

**Electronic supplementary material:**

The online version of this article (doi:10.1186/s40793-016-0159-5) contains supplementary material, which is available to authorized users.

## Introduction

*Flavobacteria*/*Cytophagia* have been frequently observed in aquatic and soil habitats [1–3] and play a major role in polysaccharide decomposition [2, 4, 5]. Type strains of the genus *Flavobacterium* have been isolated from many different habitats such as fresh water, sea ice and soil, and some *Flavobacterium* strains are pathogenic to humans and animals [2, 6]. Strain GH29-5^T^ (= DSM 17707^T^ = CIP 109901^T^ = KACC 11423^T^) is the type strain of *Flavobacterium suncheonense* [2, 7], which belongs to *Flavobacteriaceae* [8]. *F. suncheonense* GH29-5^T^ was isolated from greenhouse soil in Korea [10]. *Flavobacterium johnsoniae* UW101^T^, a well studied model organism, was as well isolated from soil [11, 12] and harbors a considerable number of CAZymes and PULs [13]. Thus, an investigation of the genome of strain GH29-5^T^ will give further insights into the variety of CAZymes and the polysaccharide decomposition potential of this microrganism.

Here we present the set of carbohydrate active enzymes, polysaccharide utilization loci and peptidases of *F. suncheonense* GH29-5^T^, together with a set of phenotypic features and the description and annotation of the high-quality draft genome sequence from a culture of DSM 17707^T^.

## Organism information

### Classification and features

**Fig. 1:**
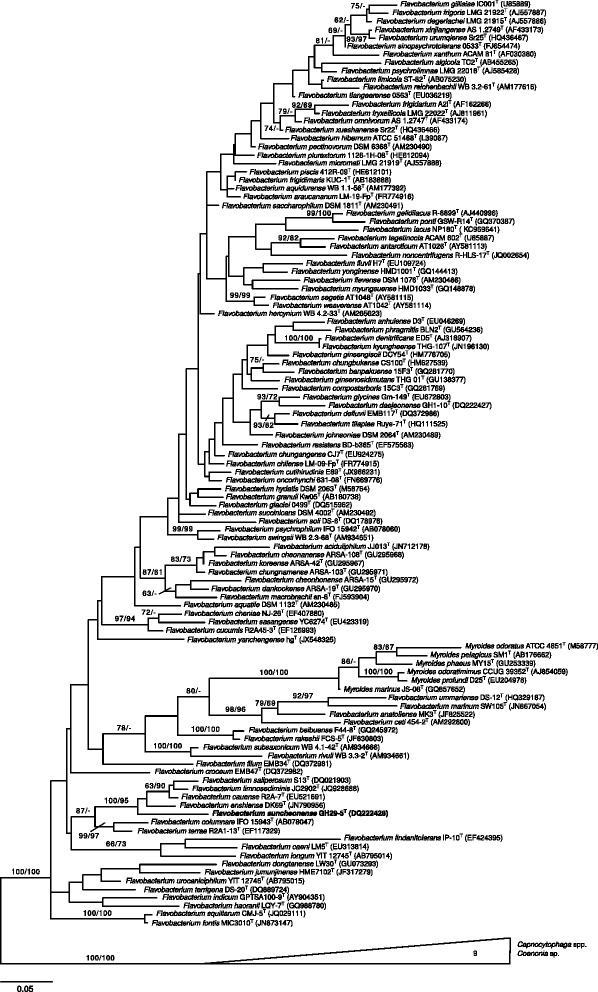
Phylogenetic tree of the genus *Flavobacterium* and its most closely related genus *Capnocytophaga*. Modified from Hahnke et al. [68]. In short: the tree was inferred from 1254 aligned characters of the 16S rRNA gene sequence under the maximum likelihood (ML) criterion. The branches are scaled in terms of the expected number of substitutions per site. Numbers adjacent to the branches are support values from 1000 ML bootstrap replicates (*left*) and from 1000 maximum-parsimony bootstrap replicates (*right*) if larger than 60 %

The sequence of the single 16S rRNA gene copy in the genome is identical with the previously published 16S rRNA gene sequence (DQ222428). Figure 1 shows the phylogenetic neighborhood of *F. suncheonense* GH29-5^T^ inferred from a tree of 16S rRNA gene sequence, as previously described [14]. The next related type species are *F. cauense* R2A-7^T^ (EU521691), *F. enshiense* DK69^T^ (JN790956), *F. limnosediminis* JC2902^T^ (JQ928688) and *F. saliperosum* S13^T^ (DQ021903) with less than 95.9 % 16S rRNA gene identity. The 16S rRNA gene sequence of strain GH29-5^T^ has an identity of only 93.9 % with *F. aquatile*DSM 1132^T^ (AM230485).

The 16S rRNA gene sequence of *F. suncheonense* GH29-5^T^ was compared with the Greengenes database [15]. Considering the best 100 hits, 99 sequences belonged to *Flavobacterium* and one sequence to *Cytophaga* sp. (X85210). Among the most frequent keywords within the labels of environmental samples were 40.4 % marine habitats (such as marine sediment, deep sea, seawater, whale fall, diatom/phytoplankton bloom, Sargasso Sea, sponge, sea urchin, bacterioplankton), 12.3 % soil habitats (such as rhizosphere, grassland, compost), 11.6 % freshwater habitats (such as lake, riverine sediment, groundwater), 8.9 % cold environments (such as Antarctic/Artic seawater, lake ice or sediment), but also 2.7 % wastewater habitats. Interestingly, environmental 16S rRNA gene sequences with 99 % sequence identity with *F. suncheonense* GH29-5^T^ were clones from wetland of France (KC432449) [16] and an enrichment culture of heterotrophic soil bacteria from the Netherlands (JQ855723), and with 98 % sequence identity to a soil isolate from Taiwan (DQ239767).

**Fig. 2:**
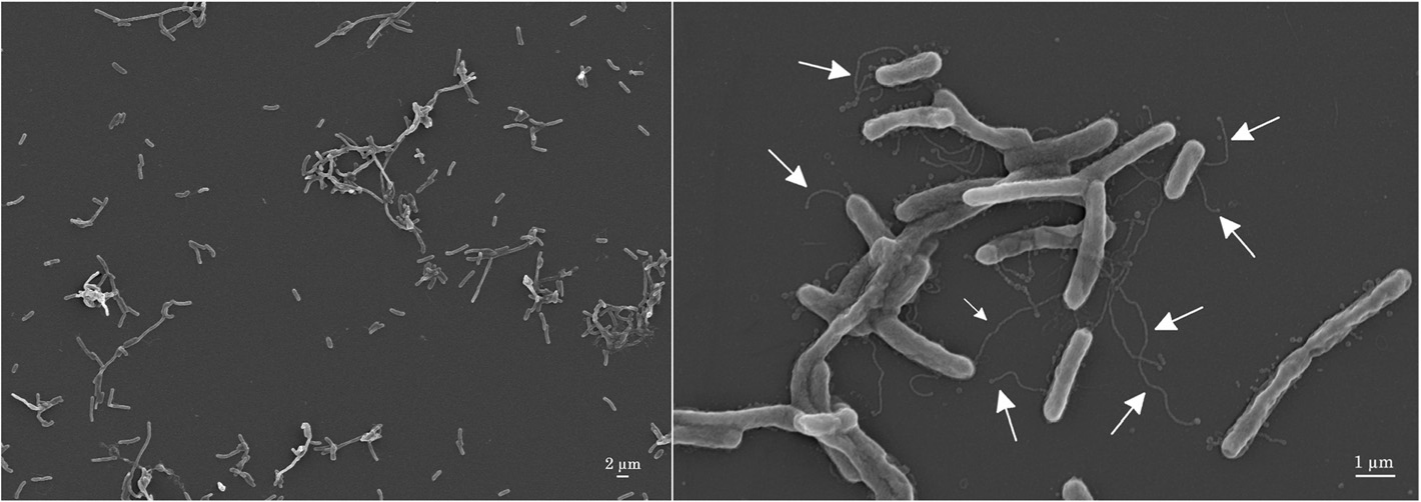
Scanning-electron micrograph of *F. suncheonense* GH29-5^T^ (DSM 17707^T^) showing appendages 50–80 nm in diameter and 0.5–8 μm in length (*arrows*)

As described for *Flavobacterium* [17], *F. suncheonense* GH29-5^T^ stains are Gram-negative (Table 1). The colonies are convex, round and yellow, but flexirubin-type pigments are absent and gliding motility was not observed [10]. The strain is positive for the catalase and oxidase tests [10], as are most members of the genus *Flavobacterium* [6]. Cells divide by binary fission, possess appandages and occur either as single rod shaped cells, with 0.3 μm in width and 1.5–2.5 μm in length, or as filaments (Fig. 2).

**Table 1:** Classification and general features of *F. suncheonense* GH29-5^T^ in accordance with the MIGS recommendations [59], as developed by [60], List of Prokaryotic names with Standing in Nomenclature [61] and the Names for Life database [62]

MIGS ID	Property	Term	Evidence code
	Current classification	Domain: *Bacteria*	TAS [12]
		Phylum: *Bacteroidetes*	TAS [63, 64]
		Class: ‘*Flavobacteriia’*	TAS [65, 66]
		Order: *Flavobacteriales*	TAS [9, 67]
		Family: *Flavobacteriaceae*	TAS [8, 9]
		Genus: *Flavobacterium*	TAS [6, 68]
		Species: *Flavobacterium suncheonense*	TAS [10]
		Type strain: GH29-5^T^	TAS [10]
	Gram-stain	Negative	TAS [10]
	Cell shape	rod-shaped	TAS [10]
	Motility	Nonmotile	TAS [10]
	Sporulation	non-spore forming	NAS
	Temperature range	mesophilic (15–37 °C)	TAS [10]
	Optimum temperature	16–24 °C	TAS [10]
	pH range; Optimum	6–8,	TAS [10]
	Carbon source	Carbohydrates, peptides	TAS [10]
	Energy source	chemoheterotroph	TAS [10]
MIGS-6	Habitat	greenhouse soil	TAS [10]
MIGS-	Salinity	0–1 % NaCl, 0 % NaCl	TAS [10]
MIGS-22	Oxygen requirement	aerobe	TAS [10]
MIGS-15	Biotic relationship	free-living	TAS [10]
MIGS-14	Pathogenicity	unknown	TAS [69]
	Biosafety level	1	TAS [69]
MIGS-4	Geographic location	Suncheon City, South Korea	TAS [10]
MIGS-5	Sample collection	2005	TAS [10]
MIGS-	Latitude	34.954	TAS [10]
MIGS-4.2	Longitude	127.483	TAS [10]
MIGS-4.4	Altitude	not reported	TAS [10]

Evidence codes are from the Gene Ontology project [18]

Evidence codes - *IDA* inferred from direct assay (first time in publication); *TAS* traceable author statement (i.e., a direct report exists in the literature); *NAS* non-traceable author statement (i.e., not directly observed for the living, isolated sample, but based on a generally accepted property for the species, or anecdotal evidence)

*F. suncheonense* GH29-5^T^ grows between 15 °C and 37 °C, pH 6 and 8 and in media with up to 1 % NaCl [10], with optimal growth at pH 7.0 and without NaCl [7]. Strain GH29-5^T^ decomposes gelatin and casein, but not starch, carboxymethyl cellulose, agar, alginate, pectin, chitin, aesculin and DNA [10]. Strain GH29-5^T^ produces H_2_S and neither reduces nitrate nor produces indole or ferments glucose [10]. Moreover, strain GH29-5^T^ does not utilize arabinose, mannose, N-acetyl-D-glucosamine, maltose, gluconate, caprate, adipate, malate, citrate and phenylacetate [19]. Strain GH29-5^T^ possesses alkaline phosphatase, esterase C4, esterase lipase C8, leucine arylamidase, valine arylamidase, acid phosphatase, naphthol-AS-BI-phosphohydrolase and N-acetyl-*β*-glucosaminidase, but has no lipase C14, cystine arylamidase, trypsin, *α*-chymotrypsin, *α*-galactosidase, *β*-galactosidase, *β*-glucuronidase, *α*-glucosidase, *β*-glucosidase, *α*-mannosidase, *α*-fucosidase and urease activity [10].

#### Chemotaxonomic data

The major cellular fatty acids are *iso*-C_15 : 0_ (29.9 %), iso-C_17 : 0_ 3-OH (17.7 %), iso-C_15 : 1_ G (12.0 %) and iso-C_15 : 0_ 3-OH (11.1 %) and MK-6 is the sole quinone [10], as common in *Flavobacterium* [6]. Besides phosphatidylethanol-amine, several unidentified lipids, aminolipids and aminophospholipids were observed in strain GH29-5^T^ [7]. The DNA G + C content was reported to be 39.0 mol % [10].

## Genome sequencing information

### Genome project history

This strain was selected for sequencing on the basis of its phylogenetic position [20, 21], and is part of Genomic Encyclopedia of Type Strains, Phase I: the one thousand microbial genomes (KMG) project [22], a follow-up of the Genomic Encyclopedia of *Bacteria* and *Archaea* (GEBA) pilot project [23], which aims at sequencing key reference microbial genomes and generating a large genomic basis for the discovery of genes encoding novel enzymes [24]. KMG-I is the part of the “Genomic Encyclopedia of *Bacteria* and *Archaea*: sequencing a myriad of type strains initiative” [25] and a Genomic Standards Consortium project [26]. The genome project is deposited in the Genomes OnLine Database [27] and the permanent draft genome sequence is deposited in GenBank. Sequencing, finishing and annotation were performed by the DOE-JGI using state-of-the-art sequencing technology [28]. A summary of the project information is shown in Table 2.

**Table 2:** Project information

MIGS ID	Property	Term
MIGS 31	Finishing quality	Level 2: High-Quality Draft
MIGS-28	Libraries used	Illumina Std shotgun library
MIGS 29	Sequencing platforms	Illumina, Illumina HiSeq 2000, Illumina HiSeq 2500
MIGS 31.2	Fold coverage	115.3x
MIGS 30	Assemblers	Velvet v. 1.1.04; ALLPATHS v. r41043
MIGS 32	Gene calling method	Prodigal, GenePRIMP, IMG-ER
	Locus Tag	G498
	Genbank ID	AUCZ00000000
	GenBank Date of Release	12-DEC-2013
	GOLD ID	Gp0013510
	BIOPROJECT	PRJNA185581
MIGS 13	Source Material Identifier	DSM 17707
	Project relevance	Tree of Life, GEBA-KMG

### Growth conditions and genomic DNA preparation

A culture of GH29-5^T^ (DSM 17707) was grown aerobically in DSMZ medium 830 (R2A Medium) [29] at 28 °C. Genomic DNA was isolated using a Jetflex Genomic DNA Purification Kit (GENOMED 600100) following the standard protocol provided by the manufacturer. DNA is available from the DSMZ through the DNA Bank Network [30].

### Genome sequencing and assembly

The draft genome of strain GH29-5^T^ was generated using the Illumina technology [31]. An Illumina Std. shotgun library was constructed and sequenced using the Illumina HiSeq 2000 platform which generated 9,392,462 reads totaling 1408.9 Mbp (Table 3). All general aspects of library construction and sequencing performed at the DOE-JGI can be found at [32]. All raw sequence data were passed through DUK, a filtering program developed at DOE-JGI, which removes known Illumina sequencing and library preparation artifacts (Mingkun L, Copeland A, Han J: DUK. *unpublished* 2011). The following steps were performed for assembly: (1) filtered reads were assembled using Velvet [33], (2) 1–3 Kbp simulated paired-end reads were created from Velvet contigs using wgsim [34], (3) Sequence reads were assembled with simulated read pairs using Allpaths–LG [35]. Parameters for assembly steps were: 1) Velvet (“velveth 63 -shortPaired” and “velvetg -very_clean yes -exportFiltered yes -min_contig_lgth 500 -scaffolding no -cov_cutoff 10”), (2) wgsim (“wgsim -e 0–1 100–2 100 -r 0 -R 0 -X 0”) (3) Allpaths–LG (“PrepareAllpathsInputs: PHRED_64 = 1 PLOIDY = 1 FRAG_COVERAGE = 125 JUMP_COVERAGE = 25 LONG_JUMP_COV = 50” and “RunAllpathsLG THREADS = 8 RUN = std shredpairs TARGETS = standard VAPI_WARN_ONLY = True OVERWRITE = True”). The final draft assembly contained 57 contigs in 54 scaffolds. The total size of the genome is 2.9 Mbp and the final assembly is based on 331.3 Mbp of data, which provides a 114.2x average coverage of the genome.

**Table 3:** Genome statistics

Attribute	Value	% of Total
Genome size (bp)	2,880,663	100.0
DNA coding (bp)	2,622,751	91.1
DNA G + C (bp)	1,165,575	40.5
DNA scaffolds	54	
Total genes	2821	100.0
Protein coding genes	2739	97.1
RNA genes	82	2.9
Pseudo genes	0	0.0
Genes in internal clusters	125	4.43
Genes with function prediction	1916	67.92
Genes assigned to COGs	1439	51.01
Genes with Pfam domains	2020	71.61
Genes with signal peptides	348	12.34
Genes with transmembrane helices	631	22.37
CRISPR repeats	0	

### Genome annotation

Genes were identified using Prodigal [36] as part of the DOE-JGI genome annotation pipeline [37], followed by manual curation using the DOE-JGI GenePRIMP pipeline [38]. The predicted CDSs were translated and used to search the National Center for Biotechnology Information non-redundant database, UniProt, TIGR-Fam, Pfam, PRIAM, KEGG, COG, and InterPro database. These data sources were combined to assert a product description for each predicted protein. Additional gene prediction analysis and functional annotation was performed within the IMG-ER platform [39].

## Genome properties

The assembly of the draft genome sequence consists of 54 scaffolds amounting to 2,880,663 bp. The G + C content is 40.5 % (Table 3) which is 1.5 % higher than previously reported by Kim et al. [10] and thus shows a difference that surpasses the maximal range among strains belonging to the same species [40]. Of the 2821 genes predicted, 2739 were protein-coding genes, and 82 RNAs. The majority of the protein-coding genes (69.2 %) were assigned a putative function while the remaining ones were annotated as hypothetical proteins. The distribution of genes into COG functional categories is presented in Table 4.

**Table 4:** Number of genes associated with the general COG functional categories

Code	Value	% age	Description
J	178	11.5	Translation, ribosomal structure and biogenesis
A	–	–	RNA processing and modification
K	83	5.3	Transcription
L	76	4.9	Replication, recombination and repair
B	1	0.1	Chromatin structure and dynamics
D	24	1.5	Cell cycle control, cell division, chromosome partitioning
Y	–	–	Nuclear structure
V	44	2.8	Defense mechanisms
T	53	3.4	Signal transduction mechanisms
M	165	10.6	Cell wall/membrane/envelope biogenesis
N	10	0.6	Cell motility
Z	–	–	Cytoskeleton
W	–	–	Extracellular structures
U	15	1.0	Intracellular trafficking, and secretion
O	93	6.0	Posttranslational modification, protein turnover, chaperones
C	84	5.4	Energy production and conversion
G	51	3.1	Carbohydrate transport and metabolism
E	109	7.1	Amino acid transport and metabolism
F	62	4.0	Nucleotide transport and metabolism
H	99	6.4	Coenzyme transport and metabolism
I	77	5.0	Lipid transport and metabolism
P	74	4.8	Inorganic ion transport and metabolism
Q	29	1.9	Secondary metabolites biosynthesis, transport and catabolism
R	131	8.4	General function prediction only
S	83	5.3	Function unknown
–	1382	49.0	Not in COGs

## Insights from the genome sequence

### Comparative genomics

We conducted a comparative genomics analysis of *F. suncheonense* (AUCZ00000000) with a selection of closely related (according to 16S rRNA gene sequence similarities) *Flavobacterium* type strains, i.e., *F. enshiense* (AVCS00000000), *F. cauense* (AVBI00000000), *F. saliperosum* (AVFO00000000) and *F. columnare* (CP003222) and the type species *F. aquatile* (JRHH00000000). The genome sizes of the five type strains were 3.1 Mbp on average with the biggest difference of 0.5 Mbp between the genomes of *F. suncheonense* and *F. saliperosum*, on the one hand, and *F. enshiense*, on the other hand. Genome sizes wer*e* 3.1 Mbp (*F. cauense*)*,* 3.2 Mbp (*F. columnare*), 3.4 Mbp (*F. enshiense*), 2.9 Mbp (*F. suncheonense*) and 2.9 Mbp (*F. saliperosum*). However, since these genomes have not yet been sequenced completely, their sizes might slightly change in the future.

An estimate of the overall similarity between *F. suncheonense* and the five reference strains was conducted using the Genome-to-Genome Distance Calculator (GGDC 2.0) [41, 42]. It reports model-based DDH estimates (digital DDH or dDDH) along with their confidence intervals [42], which allow for genome-basted species delineation and genome-based subspecies delineation. The recommended distance formula 2 is robust against the use of incomplete genome sequences and is thus especially suited for this dataset.

The result of this comparison is shown in Table 5 and yields dDDH of below 22 % throughout, which confirms the expected status of distinct species. Furthermore, the G + C content was calculated from the genome sequences of the above strains and their pairwise differences were assessed with respect to *F. suncheonense*. Differences were 2.4 % (*F. cauense*), 2.8 % (*F. enshiense*), 1 % (*F. saliperosum*), 9.1 % (*F. columnare*) and 8.3 % (*F. aquatile*). These differences confirm the status of distinct species, because, if computed from genome sequences, these differences can only vary up to 1 % within species [40].

**Table 5:** Pairwise comparison using the GGDC (Genome-to-Genome Distance Calculator) of *F. suncheonense* with a selection of currently available *Flavobacterium* genomes, *F. enshiense* (AVCS00000000), *F. cauense* (AVBI00000000), *F. saliperosum* (AVFO00000000) and *F. columnare* (CP003222), plus the type species *F. aquatile* (JRHH00000000)

*F. suncheonense*versus	% dDDH	% C.I. dDDH	HSP length/total length [%]	Identities HSP/length [%]	Identities/total length [%]
*F. aquatile*	18.7	2.6	4	76	3
*F. cauense*	21.2	3.0	45	79	36
*F. columnare*	20.9	2.6	4	79	3
*F. enshiense*	20.2	2.9	29	78	23
*F. saliperosum*	21.0	3.0	41	79	33

Digital DDH values (dDDH) and the respective confidence intervals (C.I.) are specified for GGDC's recommended formula 2. The columns “HSP length / total length [%]”, “identities / HSP length [%]” and “identities / total length [%]” list similarities as calculated from the intergenomic distances, which were also reported by the GGDC (Formulae 1–3)

### Gliding motility

McBride and Zhu [43] described the diversity of genes involved in gliding motility among members of phylum *Bacteroidetes*. The machinery for gliding motility is composed of adhesin-like proteins, the type IX secretion system, and additional proteins [43]. Even though strain GH29-5^T^ was never observed to glide [10], all necessary genes for gliding motility were identified in its genome (Table 6).

**Table 6:** Gliding motility-related genes in strain GH29-5^T^ compared to genes in *Flavobacterium* strains studied by McBride and Zhu [43]

	*F. suncheonense* GH29-5^T^	*F. rivuli* DSM 21788^T^	*F. johnsoniae* ATCC 17061^T^
locus tag prefix	G498_RS01	F565_ RS01	Fjoh_
Gliding motility	–	–	+
Adhesin-like			
*remA*	00716	–	0808
*remB*	01803	–	1657
*sprB*	+^b^	–	0979
ATP-binding cassette transporter			
*gldA*	02505	05270	1516
*gldF*	02374	00760	2722
*gldG*	02375	00765	2721
Additional proteins			
*gldB* ^a^	00808	13390	1793
*gldC*	00807	13385	1794
*gldD* ^a^	01936	18865	1540
*gldE*	00405	18860	1539
*gldH* ^a^	02655	10515	0890
*gldJ* ^a^	00438	11845	1557
Peptidoprolyl isomerase (‘*Flavobacteriia’*, protein folding)			
*gldI*	01009	08180	2369
Type IX secretion system (secretion of RemA/RemB)			
*gldK* ^a^	00758	18605	1853
*gldL* ^a^	00757	18600	1854
*gldM* ^a^	00756	18595	1855
*gldN* ^a^	00755	18590	1856
*sprA* ^a^	01807	06065	1653
*sprE* ^a^	02154	19150	1051
*sprT* ^a^	02545	05475	1466

^a^essential gliding motility genes after McBride and Zhu [43]

^b^partial gene sequences, located at the beginning of AUCZ00000022 and at the end of AUCZ00000002

### Carbohydrate active enzymes and peptidases

Cottrell and Kirchman [44] showed that members of the *Cytophaga-Flavobacteria* group preferentially consume polysaccharides and proteins rather than amino acids. This phenotypic feature was attributed by Fernández-Gómez et al. [4] to higher numbers of peptidases and additionally higher numbers of glycoside hydrolases and carbohydrate-binding modules in the genomes of *Bacteroidetes* compared to other bacteria. *F. suncheonense* GH29-5^T^ was isolated from greenhouse soil, hydrolyzes casein and gelatin, but did not utilize any of the tested saccharides [10, 19]. Therefore, we compared the predicted CDS against the CAZyme [45, 46] and dbCAN [47] database. The CAZyme annotation (Additional file 1, Table S1) was a combination of RAPSearch2 search [48, 49] and HMMER scanning [50] as described in Hahnke et al. [14]. The genome of strain GH29-5^T^ comprised a small number of carbohydrate active enzymes (49) including 36 glycosyl transferases, nine glycoside hydrolases, four carbohydrate binding modules and six carbohydrate esterases (Table 7). Furthermore, sulfatases were suggested as important enzymes for the metabolic potential of *Bacteroidetes* to degrade sulfated algae polysaccharides such as carrageenan, agarans and fucans. Only, three sulfatases were identified in the genome of strain GH29-5^T^ (Additional file 1, Table S2).

**Table 7:** Carbohydrate active enzymes (CAZy) in the genome of strain GH29-5^T^

CAZy family	GH2	GH3	GH20	GH23	GH25	GH73	GH92
Counts	1	1	1	2	1	1	1
CAZy family	GH^a^		CBM50	CBM^a^			
Counts	1		3	1			
CAZy family	GT2	GT4	GT5	GT9	GT19	GT28	GT30
Counts	14	11	1	2	1	1	1
CAZy family	GT51	GT56					
Counts	4	1					
CAZy family	CE4	CE11	CE14	CE^a^		AA1	AA^a^
Counts	2	1	2	1		1	1

^a^genes attributed to an enzyme class, but not to a family

### Polysaccharide utilization loci

CAZymes of *Flavobacteria* that are suggested to be involved in polysaccharide decomposition are frequently observed to be organized in gene clusters. Such polysaccharides-utilization loci (PULs) consist of a TonB-dependent receptor, a SusD-like protein and carbohydrate active enzymes [51, 52]. In strain GH29-5^T^ five TonB-dependent transporters were identified of which G498_00119, G498_01595, G498_02575 were associated to siderophores and G498_00706, G498_00915 were associated with a SusD-like protein. The gene cluster up-stream of the TonB-dependent transporter G498_00706 comprised five hypothetical proteins.

### Peptidases

The MEROPS annotation was carried out by searching the sequences against the MEROPS 9.10 database [53] (access date: 2014.10.16, version: pepunit.lib) as described in Hahnke et al. [14]. The genome of strain GH29-5^T^ comprised 117 identified peptidase genes (or homologues), mostly serine peptidases (S, 50), metallo peptidases (M, 50) and cysteine peptidases (C, 14) (Table 8, Additional file 1: Tables S3 and S4). Hence, the low number of carbohydrate active enzymes and the high number of peptidases in the genome of strain GH29-5^T^ reflects its currently known substrate range being proteins rather than saccharides.

**Table 8:** Peptidases and simple peptidase inhibitors in the genome of strain GH29-5^T^

Peptidase	M01	M03	M12	M13	M14	M16	M20	M23	M24
Counts	4	1	2	2	5	2	3	6	2
Peptidase	M28	M36	M38	M41	M42	M43	M48	M50	M61
Counts	3	1	4	1	1	2	1	1	1
Peptidase	M79	M90							
Counts	1	1							
Peptidase	S01	S06	S08	S09	S12	S14	S16	S24	S26
Counts	1	1	3	16	5	1	3	1	1
Peptidase	S33	S41	S46	S49	S51	S54	S66		
Counts	6	3	2	1	1	4	1		
Peptidase	C01	C25	C26	C40	C44	C45	C56		
Counts	1	1	5	2	3	1	1		
Peptidase	N11		T02		U32	U73		A08	A28
Counts	1		1		4	1		1	1
Inhibitor	I39	I87							
Counts	4	1							

## Conclusions

The genome of *F. suncheonense* GH29-5^T^ contains a relaltively low number of carbohydrate active enzymes in contrast to genomes of other *Flavobacteriaceae* such as *Flavobacterium branchiophilum* [54], *Flavobacterium rivuli* [14], *Formosa agariphila* [55], *Polaribacter* [4, 56], ‘*Gramella forsetii*’ [57] and *Zobellia galactanivorans* [17]. This is surpising, since greenhouse soil might be a rich source of plant litter. McBride et al. [13] described the genome features of *Flavobacterium johnsoniae* UW101^T^, a bacterium that was as well isolated from soil [11, 58]. Both the genomes of *F. johnsoniae* UW101^T^ and *F. suncheonense* GH29-5^T^ have an almost equal number of 31 and 39 peptidases per Mbp, respectively. The genomes, however, differ remarkably in the number of CAZymes, with 47 genes per Mbp in the genome of *F. johnsoniae* UW101^T^ and only 18 genes per Mbp in the genome of *F. suncheonense* GH29-5^T^. Thus, this small set of CAZymes contributes only little to a pool of enzymes, which might be essential for a *Flavobacterium* to feed on soil components.

A systematic collection of genome sequences, such as GEBA [23] and KMG-1 [22], will provide the scientific community with the possibility for a systematic discovery of genes encoding for novel enzymes [24] and support microbial taxonomy. In addition, genome sequences also provide further taxonomically useful information such as the G + C content [40], which, as seen in this report might significantly differ from the values determined with traditional methods.

Based on the observed large difference in the DNA G + C content and the additional information on cell morphology obtained in this study, an emended description of *F. suncheonense* is proposed.

## Emended description of *F. suncheonense* GH29-5^T^ Kim et al. 2006 emend. Dong et al. 2013

The description of *Flavobacterium suncheonense* is as given by Kim et al. [10] and Dong et al. [7], with the following modifications: the DNA G + C content is 40.5 mol%, and amendments: possesses appendages of 50–80 nm in diameter and 0.5–8 μm in length.

## Additional file

Additional file 1:
**Table S1**. Carbohydrate active enzymes (CAZymes) in the genome of *F. suncheonense* GH29-5^T^. **Table S2**. Sulfatases in the genome of *F. suncheonense* GH29-5^T^. **Table S3**. Peptidases or homologues in the genome of *F. suncheonense* GH29-5^T^. **Table S4**. Simple peptidases inhibitors in the genome of *F. suncheonense* GH29-5^T^. (DOCX 497 kb)

## Abbreviations

DOE: Department of Energy
EMBL: European molecular biology laboratory
GEBA: Genomic encyclopedia of *Bacteria* and *Archaea*
JGI: Joint Genome Institute
IMG-ER: Integrated microbial genomes – expert review
KMG: One thousand microbial genomes project
RDP: Ribosomal database project (East Lansing, MI, USA)
