# Supplementary material for: High-quality draft genome sequence of Flavobacterium suncheonense GH29-5T (DSM 17707T) isolated from greenhouse soil in South Korea, and emended description of Flavobacterium suncheonense GH29-5T
Source: Stand Genomic Sci. 2016 Jun 16;11:42. doi: 10.1186/s40793-016-0159-5 (PMC4910214; doi:10.1186/s40793-016-0159-5)
Supplement: Additional file 1: — Table S1. Carbohydrate active enzymes (CAZymes) in the genome of F. suncheonense GH29-5T. Table S2. Sulfatases in the genome of F. suncheonense GH29-5T. Table S3. Peptidases or homologues in the genome of F. suncheonense GH29-5T. Table S4. Simple peptidases inhibitors in the genome of F. suncheonense GH29-5T. (DOCX 497 kb) [file 40793_2016_159_MOESM1_ESM.docx]

High-quality draft genome sequence of *Flavobacterium suncheonense* GH29-5^T^ (DSM 17707^T^) isolated from greenhouse soil in South Korea

Nisreen Tashkandy [1](http://standardsingenomics.org/index.php/sigen/article/view/508/1119#aff2)^*^, Sari Sabban [1](http://standardsingenomics.org/index.php/sigen/article/view/508/1119#aff2), Mohammad Fakieh[1](http://standardsingenomics.org/index.php/sigen/article/view/508/1119#aff2), Richard L. Hahnke^2^, Jan P. Meier-Kolthoff^2^, Sixing Huang^2^, Brian J. Tindall^2^, Manfred Rohde^3^, Mohammed N. Baeshen^1,4^ , Nabih A. Baeshen^1,4^ , Alla Lapidus[5](http://standardsingenomics.org/index.php/sigen/article/view/508/1119#aff2)^,6^, Alex Copeland[7](http://standardsingenomics.org/index.php/sigen/article/view/508/1119#aff2), Manoj Pillay^8^, T.B.K. Reddy^7^, Marcel Huntemann^7^, Amrita Pati^7^, Natalia Ivanova^7^,  Victor Markowitz^8^, Tanja Woyke^7^[,](http://standardsingenomics.org/index.php/sigen/article/view/sigs.3216895/813#aff2) Hans-Peter Klenk^9¥^, Nikos C. Kyrpides^1,7^

#### ^1^Department of Biological Sciences, Faculty of Science, King Abdulaziz University, Jeddah, Saudi Arabia

##### ^2^Leibniz Institute DSMZ – German Collection of Microorganisms and Cell Cultures, Braunschweig, Germany

#### ^3^HZI – Helmholtz Centre for Infection Research, Braunschweig, Germany

#### ^4^Center of Nanotechnology, King Abdulaziz University, Jeddah, Saudi Arabia

##### ^5^Dobzhansky Center for Genome Bioinformatics, St. Petersburg State University, St. Petersburg, Russia

##### ^6^Algorithmic Biology Lab, St. Petersburg Academic University, St. Petersburg, Russia

##### ^7^Department of Energy Joint Genome Institute, Genome Biology Program, Walnut Creek, CA, USA

##### ^8^Biological Data Management and Technology Center, Lawrence Berkeley National Laboratory, Berkeley, CA, USA

##### ^9^School of Biology, Newcastle University, Newcastle upon Tyne, United Kingdom

^*^Corresponding author: Nisreen Tashkandy ntashkandy@yahoo.com

^¥^formerly affiliation 2

***Supporting Information Text***

**Table S1.** Carbohydrate active enzymes (CAZymes) in the genome of *F. suncheonense* DSM 17707^T^.

| **Locus tag** | **CAZy family** | **Accession No.** |
| --- | --- | --- |
| G498_RS0108510 | GH2 | WP_026980788.1 |
| G498_RS0113340 | GH3 | WP_026981613.1 |
| G498_RS0108525 | GH20 | WP_026980791.1 |
| G498_RS0100295 | GH23 | WP_026979296.1 |
| G498_RS0107505 | GH23;CBM50 | WP_026980614.1 |
| G498_RS0111560 | GH25 | WP_026981293.1 |
| G498_RS0101310 | GH73;CBM50 | WP_026979477.1 |
| G498_RS0106505 | GH92 | WP_026980437.1 |
| G498_RS0113095 | GH* | WP_026981567.1 |
|  |  |  |
| G498_RS0100345 | GT2 | WP_026979306.1 |
| G498_RS0103425 | GT2 | WP_026979869.1 |
| G498_RS0104190 | GT2 | WP_026980005.1 |
| G498_RS0104395 | GT2 | WP_026980039.1 |
| G498_RS0106190 | GT2 | WP_026980376.1 |
| G498_RS0107190 | GT2 | WP_026980559.1 |
| G498_RS0107270 | GT2 | WP_026980573.1 |
| G498_RS0110280 | GT2 | WP_026981066.1 |
| G498_RS0110470 | GT2 | WP_026981103.1 |
| G498_RS0110475 | GT2 | WP_026981104.1 |
| G498_RS0110845 | GT2 | WP_026981167.1 |
| G498_RS0111250 | GT2 | WP_026981240.1 |
| G498_RS0112320 | GT2 | WP_026981427.1 |
| G498_RS0107215 | GT4 | WP_026980564.1 |
| G498_RS0110265 | GT4 | WP_026981064.1 |
| G498_RS0111585 | GT4 | WP_026981298.1 |
| G498_RS0111595 | GT4 | WP_026981300.1 |
| G498_RS0111600 | GT4 | WP_026981301.1 |
| G498_RS0111615 | GT4 | WP_026981304.1 |
| G498_RS0111660 | GT4 | WP_026981310.1 |
| G498_RS0111825 | GT4 | WP_026981337.1 |
| G498_RS0111855 | GT4 | WP_026981342.1 |
| G498_RS0111865 | GT4 | WP_026981344.1 |
| G498_RS0113330 | GT4 | WP_026981611.1 |
| G498_RS0106870 | GT5 | WP_026980504.1 |
| G498_RS0107150 | GT9 | WP_026980552.1 |
| G498_RS0110460 | GT9 | WP_026981101.1 |
| G498_RS0103980 | GT19 | WP_026979969.1 |
| G498_RS0101730 | GT28 | WP_026979554.1 |
| G498_RS0102035 | GT30 | WP_026979611.1 |
| G498_RS0106830 | GT51 | WP_026980496.1 |
| G498_RS0109165 | GT51 | WP_026980909.1 |
| G498_RS0111870 | GT56 | WP_026981345.1 |
| Continued on next page. | | |

| **Table S1. (continued)** | | |
| --- | --- | --- |
| **Locus tag** | **CAZy family** | **Accession No.** |
| G498_RS0102840 | CBM50 | WP_026979759.1 |
| G498_RS0101915 | CBM* | WP_026979587.1 |
|  |  |  |
| G498_RS0105520 | CE4 | WP_026980253.1 |
| G498_RS0106220 | CE4 | WP_026980382.1 |
| G498_RS0112970 | CE11 | WP_026981543.1 |
| G498_RS0103220 | CE14 | WP_026979831.1 |
| G498_RS0103840 | CE14 | WP_026979946.1 |
| G498_RS0101070 | CE* | WP_026979435.1 |
|  |  |  |
| G498_RS0112470 | AA1 | WP_026981453.1 |
| * genes attributed to an enzyme class, but not to a family | | |

**Table S2.** Sulfatases in the genome of *F. suncheonense* DSM 17707^T^.

| **Locus tag** | **Name** | **Accession No.** |
| --- | --- | --- |
| G498_RS0113030 | Sulfatase | WP_026981555.1 |
| G498_RS0103805 | Sulfatase | WP_026979939.1 |
| G498_RS0110485 | Sulfatase, DUF3413 | WP_026981106.1 |
| Continued on next page. | | |

**Table S3.** Peptidases or homologues in the genome of *F. suncheonense* DSM 17707^T^.

| **Locus tag**  **(G498_)** | **MEROPS**  **family** | **Peptidase or homologue** | **Accession No.** |
| --- | --- | --- | --- |
| RS0106595 | A08 | signal peptidase II (Flavobacterium psychrophilum) | WP_026980454.1 |
| RS0104545 | A28 | family A28 unassigned peptidases | WP_026980206.1 |
|  |  |  |  |
| RS0104860 | C01 | subfamily C1B unassigned peptidases (Flavobacterium columnare) | WP_026980126.1 |
| RS0112375 | C25 | family C25 unassigned peptidases | WP_026981438.1 |
| RS0111975 | C26 | carbamoyl-phosphatesynthase,small (Flavobacterium indicum) | WP_026981366.1 |
| RS0107815 | C26 | trp1 (Flavobacterium psychrophilum) | WP_026980668.1 |
| RS0102855 | C26 | CTPsynthetase | WP_026979762.1 |
| RS0102835 | C26 | GMPsynthase (Flavobacterium columnare) | WP_026979758.1 |
| RS0100265 | C26 | dihydro-orotase (N-terminal unit) (Ailuropoda melanoleuca) | WP_026979291.1 |
| RS0113465 | C40 | family C40 unassigned peptidases (Kordia algicida) | WP_026981635.1 |
| RS0102840 | C40 | spr peptidase (Thermodesulfobacteriumsp. OPB45) | WP_026979759.1 |
| RS0110230 | C44 | AsnB protein (Flavobacteriumfrigoris) | WP_026981057.1 |
| RS0106880 | C44 | glucosamine-fructose-6-phosphate aminotransferase | WP_026980506.1 |
| RS0105040 | C44 | family C44 unassigned peptidases (Flavobacterium indicum) | WP_026980160.1 |
| RS0106175 | C45 | family C45 unassigned peptidases (Flavobacterium johnsoniae) | WP_026980373.1 |
| RS0103815 | C56 | KIAA0361 protein (Flavobacterium branchiophilum) | WP_026979941.1 |
|  |  |  |  |
| RS0113800 | M01 | family M1 unassigned peptidases | WP_026981699.1 |
| RS0107550 | M01 | family M1 unassigned peptidases (Flavobacterium columnare) | WP_026980622.1 |
| RS0106715 | M01 | family M1 unassigned peptidases (Flavobacterium indicum) | WP_026980474.1 |
| RS0101860 | M01 | family M1 unassigned peptidases (Flavobacterium johnsoniae) | WP_026979578.1 |
| RS0112245 | M03 | subfamily M3A unassigned peptidases (Flavobacterium columnare) | WP_026981413.1 |
| RS0106990 | M12 | subfamily M12B unassigned peptidases (Saprospira grandis) | WP_026980523.1 |
| RS0106980 | M12 | subfamily M12B unassigned peptidases (Chitinophaga pinensis) | WP_026980522.1 |
| RS0114345 | M13 | Zmp1 peptidase (Flavobacteriumsp. CF136) | WP_026981789.1 |
| RS0105190 | M13 | Zmp1 peptidase (Flavobacterium psychrophilum) | WP_026980189.1 |
| RS0102970 | M14 | subfamily M14B non-peptidase homologues (Dokdonia donghaensis) | WP_026979785.1 |
| RS0100425 | M14 | subfamily M14B non-peptidase homologues (Dyadobacterfermentans) | WP_026979322.1 |
| Continued on next page. | | | |

| **Table S3. (continued)** | | | |
| --- | --- | --- | --- |
| **Locus tag**  **(G498_)** | **MEROPS**  **family** | **Peptidase or homologue** | **Accession No.** |
| RS0112880 | M14 | family M14 non-peptidase homologues (Flavobacterium columnare) | WP_026981528.1 |
| RS0107730 | M14 | family M14 non-peptidase homologues (Polaribactersp. MED152) | WP_026980656.1 |
| RS0106615 | M14 | family M14 non-peptidase homologues (Flavobacterium psychrophilum) | WP_026980458.1 |
| RS0105690 | M16 | subfamily M16B non-peptidase homologues (Flavobacterium indicum) | WP_026980287.1 |
| RS0105685 | M16 | subfamily M16B non-peptidase homologues | WP_026980286.1 |
| RS0103075 | M20 | peptidase T (Flavobacterium columnare) | WP_026979806.1 |
| RS0108730 | M20 | Pep581 peptidase (Flavobacterium columnare) | WP_026980830.1 |
| RS0104125 | M20 | subfamily M20F unassigned peptidases (Flavobacterium columnare) | WP_026979993.1 |
| RS0111070 | M23 | subfamily M23B non-peptidase homologues (Caulobactersp. AP07) | WP_026981207.1 |
| RS0107040 | M23 | subfamily M23B non-peptidase homologues (Flavobacterium johnsoniae) | WP_026980531.1 |
| RS0106655 | M23 | subfamily M23B non-peptidase homologues (Flavobacterium psychrophilum) | WP_026980466.1 |
| RS0101440 | M23 | Mername-AA292 peptidase (Flavobacterium indicum) | WP_026979500.1 |
| RS0100390 | M23 | subfamily M23B unassigned peptidases (Polaribactersp. MED152) | WP_026979315.1 |
| RS0100375 | M23 | subfamily M23B unassigned peptidases (Flavobacterium johnsoniae) | WP_026979312.1 |
| RS0101935 | M24 | methionyl aminopeptidase 1 (Flavobacterium columnare) | WP_026979591.1 |
| RS0104995 | M24 | subfamily M24B unassigned peptidases (Flavobacterium johnsoniae) | WP_026980151.1 |
| RS0103095 | M28 | subfamily M28A unassigned peptidases (Flavobacterium branchiophilum) | WP_026979810.1 |
| RS0102480 | M28 | subfamily M28A unassigned peptidases (Flavobacterium columnare) | WP_026979690.1 |
| RS0103865 | M28 | subfamily M28B unassigned peptidases | WP_026979950.1 |
| RS0106055 | M36 | fungalysin (Flavobacterium psychrophilum) | WP_026980350.1 |
| RS0113645 | M38 | family M38 non-peptidase homologues (Flavobacterium columnare) | WP_026981669.1 |
| RS0110235 | M38 | family M38 non-peptidase homologues (Spirosoma linguale) | WP_026981058.1 |
| RS0104390 | M38 | family M38 non-peptidase homologues (Flavobacterium indicum) | WP_026980038.1 |
| RS0102540 | M38 | family M38 non-peptidase homologues (Flavobacterium johnsoniae) | WP_026979700.1 |
| RS0107695 | M41 | Afg3-like protein 2 | WP_026980649.1 |
| RS0113935 | M42 | family M42 unassigned peptidases | WP_026981718.1 |
| RS0113425 | M43 | ulilysin (Flavobacterium columnare) | WP_026981630.1 |
| Continued on next page. | | | |

| **Table S3. (continued)** | | | |
| --- | --- | --- | --- |
| **Locus tag**  **(G498_)** | **MEROPS**  **family** | **Peptidase or homologue** | **Accession No.** |
| RS0104365 | M43 | Mername-AA293 peptidase (Piriformospora indica) | WP_026980034.1 |
| RS0113885 | M48 | subfamily M48C unassigned peptidases (Flavobacterium columnare) | WP_026981713.1 |
| RS0101675 | M50 | family M50 non-peptidase homologues (Methanothermusfervidus) | WP_026979544.1 |
| RS0111470 | M61 | family M61 unassigned peptidases (Flavobacterium psychrophilum) | WP_026981278.1 |
| RS0113610 | M79 | family M79 unassigned peptidases (Cellulophaga algicola) | WP_026981662.1 |
| RS0101695 | M90 | family M90 unassigned peptidases (Aequorivitasublithincola) | WP_026979548.1 |
|  |  |  |  |
| RS0101150 | N11 | family N11 unassigned peptide lyases | WP_026979449.1 |
|  |  |  |  |
| RS0101055 | S01 | DegQ peptidase (Flavobacterium indicum) | WP_026979433.1 |
| RS0104340 | S06 | familys6 unassigned peptidases (Flavobacterium johnsoniae) | WP_026980029.1 |
| RS0107555 | S08 | subfamilys8A non-peptidase homologues (Flavobacterium columnare) | WP_026980623.1 |
| RS0103420 | S08 | subfamilys8A non-peptidase homologues (Fluviicola taffensis) | WP_026979868.1 |
| RS0102880 | S08 | subfamilys8A unassigned peptidases (Flavobacterium psychrophilum) | WP_026979767.1 |
| RS0113545 | S09 | oligopeptidase B (Flavobacterium indicum) | WP_026981650.1 |
| RS0107075 | S09 | prolyl oligopeptidase (Ignavibacterium album) | WP_026980538.1 |
| RS0104820 | S09 | prolyl oligopeptidase (Flavobacterium johnsoniae) | WP_026980118.1 |
| RS0112500 | S09 | prolyl tripeptidyl peptidase (Flavobacterium columnare) | WP_026981458.1 |
| RS0104015 | S09 | dipeptidyl-peptidase 4 (bacteria-type 2) (Flavobacterium columnare) | WP_026979976.1 |
| RS0104855 | S09 | dipeptidyl-peptidase 5 (Flavobacterium columnare) | WP_026980125.1 |
| RS0111405 | S09 | glutamyl endopeptidase C (Flavobacteriumfrigoris) | WP_026981266.1 |
| RS0113890 | S09 | familys9 non-peptidase homologues (Flavobacterium psychrophilum) | WP_026981714.1 |
| RS0112780 | S09 | familys9 non-peptidase homologues (Capnocytophaga ochracea) | WP_026981510.1 |
| RS0111305 | S09 | familys9 non-peptidase homologues (Flavobacterium johnsoniae) | WP_026981251.1 |
| RS0102530 | S09 | YpfH protein (Flavobacterium johnsoniae) | WP_026979698.1 |
| RS0102355 | S09 | familys9 unassigned peptidases (Flavobacterium johnsoniae) | WP_026979665.1 |
| RS0102220 | S09 | familys9 non-peptidase homologues (Zunongwangia profunda) | WP_026979644.1 |
| RS0102105 | S09 | familys9 non-peptidase homologues (Spirosoma linguale) | WP_026979621.1 |
| Continued on next page. | | | |

| **Table S3. (continued)** | | | |
| --- | --- | --- | --- |
| **Locus tag**  **(G498_)** | **MEROPS**  **family** | **Peptidase or homologue** | **Accession No.** |
| RS0101070 | S09 | familys9 non-peptidase homologues (Spirosoma linguale) | WP_026979435.1 |
| RS0100175 | S09 | familys9 non-peptidase homologues (Rhodopirellula baltica) | WP_026979274.1 |
| RS0113340 | S12 | familys12 unassigned peptidases (Flavobacterium johnsoniae) | WP_026981613.1 |
| RS0110355 | S12 | familys12 unassigned peptidases (Flavobacterium indicum) | WP_026981081.1 |
| RS0109125 | S12 | familys12 unassigned peptidases (Flavobacterium indicum) | WP_026980901.1 |
| RS0108155 | S12 | familys12 unassigned peptidases | WP_026980731.1 |
| RS0105550 | S12 | familys12 unassigned peptidases (Flavobacterium psychrophilum) | WP_026980259.1 |
| RS0102120 | S14 | peptidase Clp (type 1) | WP_026979624.1 |
| RS0113870 | S16 | Lon peptidase (type 4) | WP_026981711.1 |
| RS0112560 | S16 | familys16 non-peptidase homologues (Flavobacterium columnare) | WP_026981469.1 |
| RS0106845 | S16 | DNA repair protein RadA (Flavobacterium branchiophilum) | WP_026980499.1 |
| RS0104755 | S24 | UmuD protein | WP_026980106.1 |
| RS0111180 | S26 | subfamilys26A unassigned peptidases (Flavobacterium columnare) | WP_026981228.1 |
| RS0110900 | S33 | familys33 unassigned peptidases (Cellulophaga lytica) | WP_026981177.1 |
| RS0108445 | S33 | familys33 unassigned peptidases (Flavobacterium psychrophilum) | WP_026980780.1 |
| RS0105000 | S33 | sCO7095-type peptidase (Flavobacterium psychrophilum) | WP_026980152.1 |
| RS0102145 | S33 | familys33 non-peptidase homologues (Flavobacterium columnare) | WP_026979629.1 |
| RS0101780 | S33 | familys33 unassigned peptidases (Flavobacterium johnsoniae) | WP_026979563.1 |
| RS0100300 | S33 | familys33 unassigned peptidases (Flavobacterium johnsoniae) | WP_026979297.1 |
| RS0107535 | S41 | CtpC peptidase (Flavobacterium columnare) | WP_026980620.1 |
| RS0106115 | S41 | C-terminal processing peptidase-3 | WP_026980362.1 |
| RS0103965 | S41 | C-terminal processing peptidase-1 (Saprospira grandis) | WP_026979966.1 |
| RS0112205 | S46 | familys46 unassigned peptidases (Flavobacterium psychrophilum) | WP_026981405.1 |
| RS0102040 | S46 | familys46 unassigned peptidases | WP_026979612.1 |
| RS0102070 | S49 | BSn5_05605 g.p. (Flavobacteriumfrigoris) | WP_026979617.1 |
| RS0101880 | S51 | alpha-aspartyl dipeptidase | WP_026979582.1 |
| RS0114390 | S54 | familys54 unassigned peptidases (Flavobacterium psychrophilum) | WP_026981796.1 |
| RS0112150 | S54 | familys54 unassigned peptidases (Flavobacterium johnsoniae) | WP_026981395.1 |
| Continued on next page. | | | |

| **Table S3. (continued)** | | | |
| --- | --- | --- | --- |
| **Locus tag**  **(G498_)** | **MEROPS**  **family** | **Peptidase or homologue** | **Accession No.** |
| RS0111205 | S54 | RhoII peptidase (Flavobacterium columnare) | WP_026981232.1 |
| RS0111200 | S54 | familys54 unassigned peptidases (Flavobacterium johnsoniae) | WP_026981231.1 |
| RS0105125 | S66 | murein tetrapeptidase LD-carboxypeptidase | WP_026980177.1 |
|  |  |  |  |
| RS0108520 | T02 | N4 (beta-N-acetylglucosaminyl)-L-asparaginase (Flavobacterium indicum) | WP_026980790.1 |
|  |  |  |  |
| RS0109190 | U32 | family U32 unassigned peptidases (Elizabethkingia anophelis) | WP_026980914.1 |
| RS0109185 | U32 | collagenase (Flavobacterium columnare) | WP_026980913.1 |
| RS0105025 | U32 | family U32 unassigned peptidases (Chthonomonas calidirosea) | WP_026980157.1 |
| RS0100955 | U32 | family U32 unassigned peptidases (Rhizobium etli) | WP_026979415.1 |
| RS0102000 | U73 | small protease (Pseudomonas aeruginosa) | WP_026979604.1 |

**Table S4.** Simple peptidases inhibitors in the genome of *F. suncheonense* DSM 17707^T^.

| **Locus tag**  **(G498_)** | **MEROPS**  **family** | **Simple peptidase inhibitors** | **Accession No.** |
| --- | --- | --- | --- |
| RS0106035 | I39 | family I39 unassigned peptidase inhibitor homologues (Flavobacterium johnsoniae) | WP_026980346.1 |
| RS0102345 | I39 | family I39 unassigned peptidase inhibitor homologues (Pedobacter heparinus) | WP_026979663.1 |
| RS0101275 | I39 | family I39 unassigned peptidase inhibitor homologues (Spirosoma linguale) | WP_026979470.1 |
| RS0100155 | I39 | family I39 unassigned peptidase inhibitors (Pirellulastaleyi) | WP_026979270.1 |
| RS0107420 | I87 | family I87 unassigned peptidase inhibitors (Treponema brennaborense) | WP_026980599.1 |
